# Lytic bacteriophages targeting carbapenem-resistant Klebsiella pneumoniae isolated in a resource-limited Zambian laboratory

**DOI:** 10.1099/acmi.0.001146.v3

**Published:** 2026-09-22

**Authors:** Frank Chilombolwa Nyondo, Bwalya Daka Lalusha, Marriot Chikopa, Sody Munsaka

**Affiliations:** 1 Department of Biomedical Sciences, School of Health Sciences, University of Zambia, Ridgeway Campus, Lusaka, Zambia; 2 School of Medicine, Pathology and Microbiology, University of Zambia, Ridgeway Campus, Lusaka, Zambia

**Keywords:** antimicrobial resistance, bacteriophages, carbapenem-resistant *Klebsiella pneumoniae*, *Klebsiella pneumoniae*, multidrug resistance, phage therapy

## Abstract

*Klebsiella pneumoniae* is a major cause of healthcare-associated and community-acquired infections, increasingly complicated by multidrug resistance (MDR) and carbapenem-resistant *K. pneumoniae* (CRKp). Phage-based approaches are attractive in such settings but rely on infrastructure and expertise, which are not widely available in low-resource laboratories. Here, we describe the isolation and preliminary characterization of two lytic *K. pneumoniae* phages, Zm_01 and Zm_02, from hospital sewer sludge in Lusaka, Zambia, using only routine microbiology equipment and simple adaptations of standard protocols. Clinical *K. pneumoniae* isolates, including MDR and carbapenem-resistant (meropenem-resistant) strains, were used for enrichment, double-agar overlay plaque assays, host-range screening and determination of efficiency of plating (EOP). Zm_01, isolated on a non-MDR but hypermucoviscous host, produced small, clear plaques and exhibited efficient plating on several MDR isolates, with EOP values higher on some MDR strains than on its original host. Zm_02, isolated on an MDR strain, formed large, clear plaques with halos, consistent with capsule-degrading activity. All work was performed without access to specialized equipment, such as a spectrophotometer, a shaker incubator or standard imaging equipment. These findings demonstrate that discovery and basic phenotypic characterization of therapeutically relevant *K. pneumoniae* phages are feasible in low-resource settings and provide a practical workflow that can underpin future functional and genomic studies, as well as the development of locally tailored phage-based interventions against MDR and CRKp infections.

**Impact Statement**

Multidrug-resistant *Klebsiella pneumoniae* is a major cause of hospital-acquired infections and can be very difficult to treat when it becomes resistant to last-line antibiotics, such as carbapenems. This problem is especially serious in low- and middle-income countries, where access to newer antibiotics, advanced diagnostics and specialist research infrastructure is limited. In this study, we isolated two viruses that specifically infect and kill *K. pneumoniae* (bacteriophages) from hospital sewer sludge in Lusaka, Zambia. We showed that they can lyse several multidrug-resistant clinical isolates. All of the work was done in a routine microbiology laboratory using basic equipment, simple adaptations of standard methods and low-cost tools such as manual agitation and smartphone photography. This work adds to the growing literature on phages against carbapenem-resistant *K. pneumoniae*, but from a setting that is usually under-represented in the field. It provides a practical, stepwise workflow that other resource-limited laboratories can adopt to start local phage discovery and basic characterization. Although preliminary, these findings represent an early step towards developing locally tailored phage-based approaches to support the management of multidrug-resistant *K. pneumoniae* infections in sub-Saharan Africa.

## Data Summary

The authors confirm that all supporting data, code and protocols have been provided within the article or through supplementary data files.

## Introduction

*Klebsiella pneumoniae* is a Gram-negative enteric bacterium that colonizes humans and animals as a commensal and is widely detected in wastewater, freshwater and soil and causes pneumonia, bloodstream, urinary tract and intra-abdominal infections [1–3]. It is also a core ESKAPE pathogen associated with multidrug resistance (MDR) and healthcare-associated infections [4].

Two main clinical pathotypes are recognized: classical *K. pneumoniae*, typically associated with healthcare-associated infections and high antimicrobial resistance, and hypervirulent *K. pneumoniae* (hvKp), which causes community-acquired invasive disease and often exhibits a hypermucoviscous, capsule-rich phenotype [5]. Historically, hvKp was largely susceptible to first-line agents, but carbapenem-resistant hypervirulent strains (CR-hvKp) are increasingly reported, indicating convergence of resistance and virulence [3, 6]. Globally, carbapenem-resistant *K. pneumoniae* (CRKp) has eroded the usefulness of extended-spectrum cephalosporins, aminoglycosides and carbapenems, leaving colistin and a few older drugs as last-line options [7, 8]. In Zambia and neighbouring countries, hospital data already document MDR *K. pneumoniae* with resistance to extended-spectrum cephalosporins and aminoglycosides and reduced susceptibility to carbapenems [9–11]. The WHO and Africa-CDC [12] warn that, without effective interventions, drug-resistant infections could cause up to 10 million deaths annually by 2050, with sub-Saharan Africa disproportionately affected, underscoring the need for locally feasible alternatives and adjuncts to conventional antibiotics against CRKp.

Bacteriophages (phages) are viruses that infect bacteria; most described phages are tailed dsDNA viruses classified within the class Caudoviricetes [13]. Lytic phages and phage-derived enzymes are being explored as alternatives or adjuncts to antibiotics for CRKp [14–16]. In *K. pneumoniae*, capsule and biofilm formation are major determinants of serum resistance, immune evasion and antibiotic tolerance [17–19]. Phage-derived depolymerases and endolysins can degrade capsule and biofilm matrix, stripping these protective barriers, enhancing penetration of conventional antibiotics and increasing exposure to host immune effectors [20, 21]. Such lytic activity is typically detected and quantified using the double-agar overlay plaque assay, in which individual infectious particles produce discrete plaques within a lawn of susceptible bacteria [22], providing a culture-based platform for environmental phage discovery, purification of clonal populations and enumeration of viable phage particles [23].

Most *K. pneumoniae* phage studies have been conducted in well-resourced laboratories with advanced culture, imaging and genomic platforms, and few reports describe bacteriophage isolation and phenotypic characterization carried out entirely in low-resource African settings [24, 25]. Standard protocols often assume access to specialized equipment, and methodologies vary considerably between groups, making it difficult for laboratories without local phage expertise to identify feasible, context-appropriate workflows [26]. Against this background, there is a need for simple, robust and cost-effective approaches to isolate and partially characterize phages active against local CRKp using only routine microbiology infrastructure.

This preliminary report describes the isolation and partial characterization of two lytic *K. pneumoniae* phages (Zm_01 and Zm_02) from hospital sewer sludge in Lusaka, using adapted low-cost plaque-based methods. This work forms part of an ongoing study in which we plan to extend phenotypic, structural and genomic characterization of the phages. In the meantime, the workflow presented here offers a pragmatic starting point for laboratories with similar resource constraints that wish to initiate phage work against CRKp.

## Methods

### Bacterial isolates, propagation and string test

Five clinical *K. pneumoniae* isolates (Kp01, Kp69, Kp73, Kp88 and Kp97) were obtained from the Microbiology Laboratory at the University Teaching Hospital (UTH), a tertiary referral centre in Lusaka, Zambia. All isolates were recovered from urine specimens processed at the urine bench and were provided de-identified; no further clinical or demographic metadata were available. Isolates were identified as *K. pneumoniae* using the routine diagnostic workflow, based on colony morphology on standard media and conventional biochemical tests according to the laboratory’s standard operating procedures. No additional molecular confirmation was performed.

Isolates were subcultured on MacConkey agar and incubated overnight at 37 °C. Hypermucoviscosity was assessed using the string test as described by Hagiya *et al*. [27]. Formation of a viscous string ≥5 mm between a colony and the inoculating loop was considered positive.

### Antimicrobial susceptibility testing of bacterial isolates

Antimicrobial susceptibility testing (AST) of *K*. *pneumoniae* clinical isolates Kp01, Kp69, Kp73 and Kp88 was performed by Kirby–Bauer disc diffusion on Mueller–Hinton agar following CLSI M100 guidelines for *Enterobacterales*. The fifth isolate, Kp97, was not subjected to AST. Overnight cultures were adjusted to a 0.5 McFarland standard in 0.85% saline, swabbed to create confluent lawns and tested with tetracycline (TE), cefepime (FEP), cefotaxime (CTX), ampicillin–sulbactam (SAM), meropenem (MEM), imipenem (IMP), gentamicin (CN), amoxicillin–clavulanate (AMC) and vancomycin (VA) discs applied within 15 min of inoculation. Plates were incubated at 37 °C for 18 h, and inhibition zone diameters (mm) were interpreted as susceptible (S), intermediate (I) or resistant (R) according to CLSI breakpoints. MICs were not determined, and neither phenotypic (e.g. modified carbapenem inactivation method) nor molecular carbapenemase detection was performed; carbapenem susceptibility was therefore assessed by disc diffusion alone. MDR was defined as non-susceptibility to ≥1 agent in ≥3 antimicrobial categories [28].

### Bacteriophage isolation, purification and enumeration

Approximately 500 ml of a sewer sample was aseptically collected from the central maintenance hole serving the casualty wards of UTH, transported on ice and stored at 4 °C until processing.

Enrichment was performed according to Twest and Kropinski [29], with minor modifications. Briefly, 3 ml of sewage was diluted to 45 ml with distilled water, 5 ml of logarithmic-phase host culture was added and the mixture was incubated overnight at 37 °C. Log-phase host cultures were prepared by inoculating overnight cultures into 4 ml tryptone soy broth (TSB – HIMEDIA, Lot E00679162) and incubating at 37 °C with manual agitation every 15 min for 3 h.

After overnight enrichment, cultures were centrifuged at 4,000 ***g*** for 20 min at 4 °C, and the supernatant was passed through 0.22 µm PVDF syringe filters (Membrane Solutions, Lot 311082147) to obtain crude phage lysate, which was stored at 4 °C until use. For preliminary screening, crude lysate was spotted onto lawns of log-phase host bacteria on 1.5% tryptone soy agar (TSA – HIMEDIA, Lot E000664441), allowed to dry at room temperature and incubated at 37 °C for 18–24 h. Plates spotted with sterile broth served as negative controls and were inspected for zones of lysis.

Phage Zm_01 was isolated using the double-agar overlay method [23]. The Kp01 host was grown to logarithmic phase, and 200 µl of culture was mixed with 200 µl of 10-fold serially diluted lysate and incubated at room temperature for 15 min to allow adsorption [15]. Mixtures were added to 3 ml of 0.4% top agar (48 °C) and poured onto 1.5% TSA base plates, which were left to solidify at room temperature and then incubated at 37 °C for 24 h. Plaques were picked with a pipette tip, eluted in 1 ml of TSB at 4 °C overnight and re-plated using the same overlay method; this purification was repeated at least four times until plaques with uniform morphology were obtained. Plates yielding 30–300 plaques were counted, and titres were expressed as p.f.u. per millilitre (p.f.u. ml^−1^) using the following formula:

p.f.u. ml^−1^=number of plaques/(dilution factor×volume of diluted phage plated) [30].

The same procedure was used to isolate phage Zm_02, with plaque purification still ongoing at the time of writing.

### Host-range determination

#### Direct spot test

Preliminary host-range screening of phage Zm_01 was performed by direct spot test before quantitative efficiency of plating (EOP) assays [31]. Individual *K. pneumoniae* isolates were grown to logarithmic phase in TSB at 37 °C, and confluent lawns were prepared on TSA by swabbing each culture in three directions. Purified Zm_01 lysate (5 µl) was spotted in duplicate onto each lawn, allowed to absorb and air-dry for a few minutes, and the plates were incubated at 37 °C for 24 h. After incubation, lawns were examined for zones of lysis or growth inhibition at the spot locations and compared with control plates spotted with an equal volume of sterile broth. Isolates showing reproducible lysis were classified as preliminarily susceptible and selected for subsequent EOP determination based on plaque formation.

#### EOP assay

Isolates that showed lysis in the spot test were subjected to quantitative EOP determination using the double-agar overlay method, as described above. Logarithmic-phase cultures of the reference host (*K. pneumoniae* Kp01) and each test isolate were mixed with 10-fold serially diluted Zm_01 lysate and overlaid onto TSA base plates. After incubation at 37 °C for 18–24 h, plates containing 30–300 plaques were counted, and titres were calculated as p.f.u. ml^−1^ using the plaque enumeration formula. For each isolate, EOP was expressed as the ratio of the p.f.u. ml^−1^ on the test isolate to that on the Kp01 reference host.

## Results

### Bacterial isolates and hypermucoviscosity

Five non-duplicate clinical *K. pneumoniae* isolates (Kp01, Kp69, Kp73, Kp88 and Kp97) were recovered from routine diagnostic cultures at the UTH (Lusaka, Zambia) and identified as *K. pneumoniae* using the site’s standard culture and biochemical procedures. No additional molecular confirmation was performed. Among the five isolates examined, Kp01 demonstrated a positive string test (≥5 mm), consistent with a hypermucoviscous phenotype [27].

### Antimicrobial susceptibility profiles of clinical isolates

**Fig. 1.**
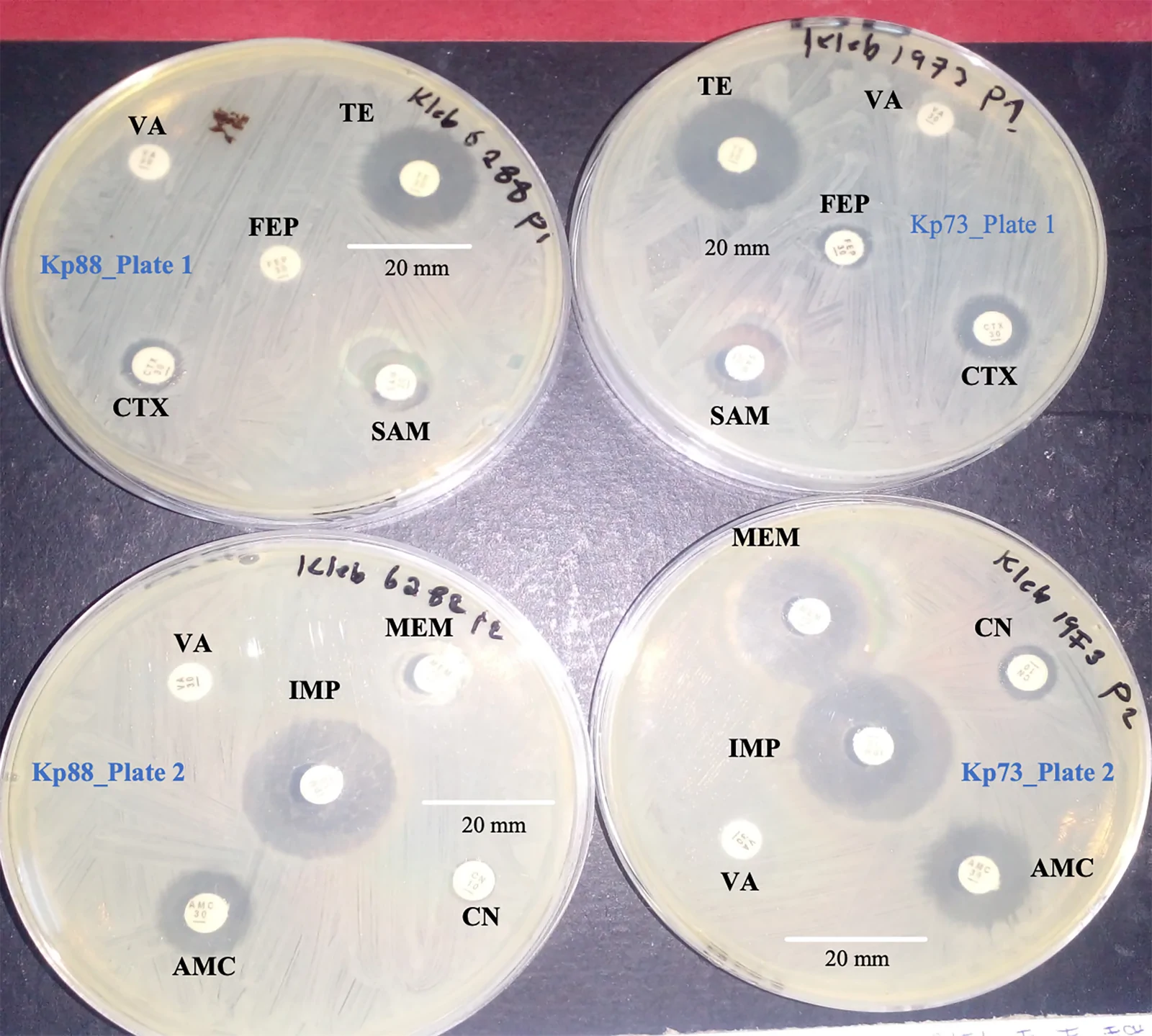
Antimicrobial susceptibility profiles of multidrug-resistant *K. pneumoniae* isolates Kp73 and Kp88 used for phage host-range testing. Representative Kirby–Bauer disc diffusion assays on Mueller–Hinton agar are shown for two independent plates per isolate (Kp88_Plate 1 and Kp88_Plate 2, left; Kp73_Plate 1 and Kp73_Plate 2, right). Discs contained VA, TE, FEP, CTX, SAM, MEM, IMP, CN and AMC, as indicated on each plate. Zones of inhibition were measured and interpreted according to current CLSI breakpoints (see Tables S1–S2), confirming multidrug-resistant phenotypes in both isolates (scale bars, 20 mm).

Disc diffusion testing was performed for four isolates (Kp01, Kp69, Kp73 and Kp88) and interpreted according to CLSI M100-Ed33 [32] breakpoints (Tables S1–S2). The resulting susceptibility profiles are summarized in Fig. 1. Three isolates (Kp69, Kp73 and Kp88) met the MDR definition (non-susceptible to ≥1 agent in ≥3 antimicrobial categories [28].

**Fig. 2.**
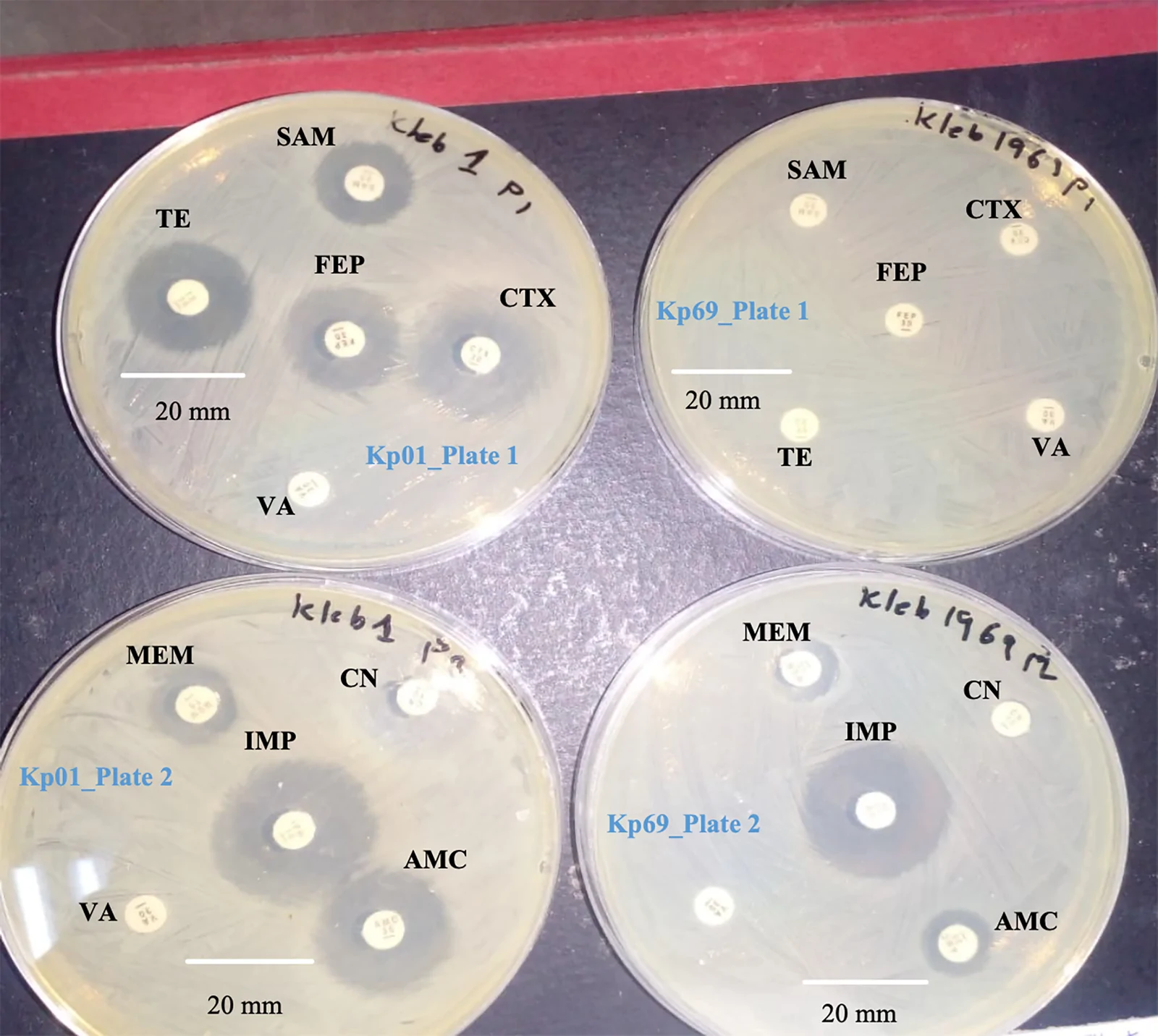
Kirby–Bauer disc diffusion susceptibility profiles of *K. pneumoniae* isolates Kp01 and Kp69. Top row: Kp01_Plate 1 (left) and Kp69_Plate 1 (right) tested with SAM, TE, FEP, CTX and VA discs. Bottom row: Kp01_Plate 2 (left) and Kp69_Plate 2 (right) tested with MEM, IMP, CN, AMC and VA discs. The reduced inhibition zones around multiple agents for Kp69 illustrate its multidrug-resistant phenotype compared with the narrower resistance profile of Kp01 (see Tables S1–S2). Scale bar=20 mm.

Kp69 was resistant to *β*-lactam/*β*-lactamase inhibitor combinations, extended-spectrum cephalosporins, MEM, TE and CN and remained susceptible only to IMP (Fig. 2). Kp73 and Kp88 also showed combined resistance to extended-spectrum cephalosporins and CN, with reduced susceptibility or resistance to MEM but preserved susceptibility to IMP and TE. In contrast, Kp01 showed a narrower profile, with resistance confined to extended-spectrum cephalosporins and MEM while remaining susceptible to IMP, aminoglycosides, *β*-lactam/*β*-lactamase inhibitor combinations and TE (Fig. 2). As expected, none of the isolates showed measurable inhibition zones around VA.

### Isolation and plaque morphology of *Klebsiella* phages Zm_01 and Zm_02

From a single sewer sample collected at the central maintenance hole serving the casualty wards, two lytic *K. pneumoniae* phages were isolated following enrichment on clinical hosts. Zm_01 was recovered using Kp01 as the propagating strain, whereas Zm_02 was isolated using the multidrug-resistant isolate Kp69.

**Fig. 3.**
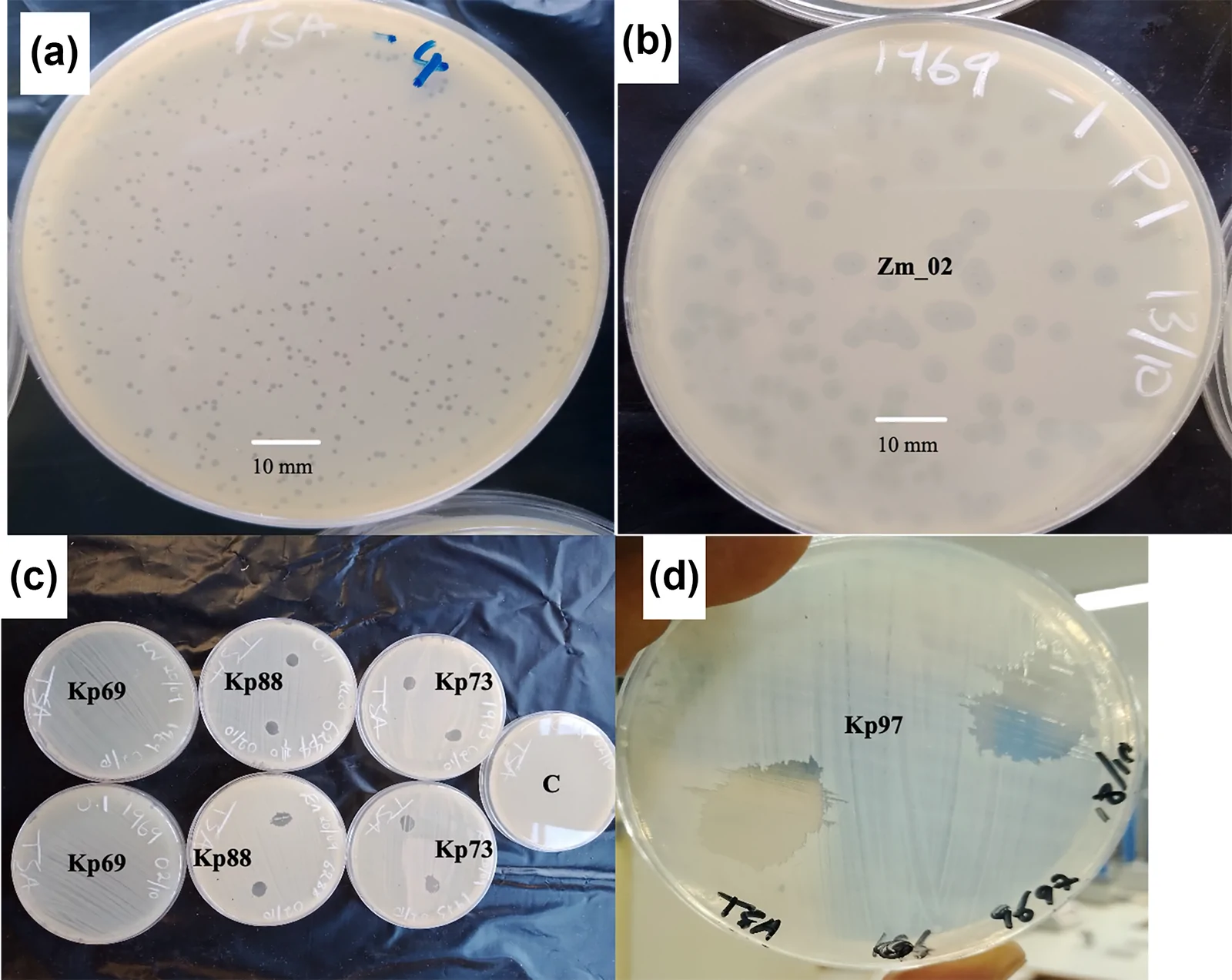
Plaque morphology and host range of *K. pneumoniae* phages Zm_01 and Zm_02. (**a**) Phage Zm_01 after five rounds of plaque purification by the double-agar overlay method using 0.4% top agar, on a 10^−4^ dilution plate with *K. pneumoniae* Kp01 as the host strain. Zm_01 produces numerous small (~0.5–1 mm), circular, sharply defined, clear plaques. (**b**) Phage Zm_02, isolated on *K. pneumoniae* Kp69 and shown at a 10^−1^ dilution, forms fewer, larger (~2–4 mm) halo-type plaques with a clear centre and diffuse outer ring. (**c**) Direct spot tests assessing host range on multidrug-resistant *K. pneumoniae* isolates Kp69, Kp73 and Kp88. Clear zones of lysis were observed on lawns of Kp73 and Kp88, whereas no lysis was detected on Kp69. (**d**) Direct spot test on clinical *K. pneumoniae* isolate Kp97, showing a distinct clearance zone at the site of phage application. Control plate shows no lysis; scale bars, 10 mm.

After five rounds of plaque purification, Zm_01 produced abundant, small (~0.5–1 mm), circular, sharply delineated, clear plaques on Kp01, consistent with a strictly lytic phenotype (Fig. 3a). Zm_02 formed fewer, larger (~2–4 mm) plaques with a clear centre and a faint halo on Kp69 (Fig. 3b), suggestive of phage-associated enzyme or depolymerase activity at the plaque periphery.

Enumeration from the final purification plates yielded an estimated titre of 3.0×10⁷ p.f.u. ml^−1^ for Zm_01 on Kp01 and 6.0×10⁹ p.f.u. ml^−1^ for Zm_02 on Kp69 (Figs S1–S2, available in the online Supplementary Material). These single-experiment titres provided the baseline for subsequent host-range and EOP assessments.

### Host-range screening by direct spot test

Purified Zm_01 lysate was screened against the clinical panel by direct spot test. Clear zones of lysis were observed on lawns of Kp73, Kp88 and Kp97, indicating susceptibility of these isolates to Zm_01. In contrast, Kp69 showed no visible lysis and retained a confluent lawn, comparable to that of the negative control spotted with sterile broth (Fig. 3c, d). No systematic host-range testing was completed for Zm_02 at the time of writing.

### EOP of Zm_01 on clinical isolates

To quantify productive infection, the EOP of Zm_01 was determined on Kp73, Kp88 and Kp97, using Kp01 as the reference host (EOP=1.0). The resulting plaque counts and calculated titres from single plating experiments are summarized in Table 1. Briefly, Zm_01 plated with markedly higher efficiency on MDR isolates Kp73 and Kp88 than on the non-MDR reference Kp01, with EOP values of 11 and 23, respectively. In contrast, plating on Kp97 was reduced (EOP 0.5), indicating lower productivity on this isolate despite apparent lysis in spot tests.

**Table 1.** EOP of *Klebsiell*a phage Zm_01 on clinical *K. pneumoniae* isolates

Isolate	Titre (p.f.u. ml^−1^)*	Relative EOP†
Kp01 (reference host)	3.0×10^7^	1.0
Kp73	3.2×10^8^	11
Kp88	7.0×10^8^	23
Kp97	1.5×10^7^	0.5

* Single plating experiment from purified Zm_01 lysate on each isolate.

†EOP calculated as (titre on test isolate)/(titre on Kp01), with Kp01 set to 1.0.

## Discussion

This short communication reports two lytic *K. pneumoniae* phages (Zm_01 and Zm_02) isolated from hospital sewer sludge and their phenotypic characterization against a clinical panel from a tertiary hospital in Lusaka, Zambia. The panel comprised three MDR isolates (Kp69, Kp73, Kp88), one non-MDR hypermucoviscous reference isolate (Kp01) resistant to extended-spectrum cephalosporins and MEM, and one further clinical isolate (Kp97) for which the susceptibility profile was not determined. Zm_01 produced small, clear plaques on Kp01 and efficiently infected the MDR isolates Kp73 and Kp88, with lower but productive infection of Kp97, whereas Zm_02, isolated on MDR Kp69, formed large halo-type plaques suggestive of capsule-degrading activity.

The observed resistance patterns are consistent with *K. pneumoniae* being a high-priority AMR pathogen in intensive care and tertiary settings [33, 34] and mirror MDR profiles already reported in Zambia and neighbouring countries [10, 11, 35]. Kp01, although not MDR by definition, was hypermucoviscous (string test ≥5 mm), a phenotype related to but distinct from hypervirulence [3]. Hypervirulence cannot be inferred from the string test alone and was not confirmed in this study, as detection of virulence biomarkers (e.g. peg-344, iroB, iucA, rmpA and rmpA2), and virulence phenotypic assays were beyond the scope of the present study [36]. Kp01 is therefore described here as hypermucoviscous, with its pathotype undetermined [6, 36]. Several MDR isolates remained susceptible to phage lysis, consistent with reports of phages retaining activity against MDR and CRKp [37].

All isolates with reduced susceptibility to MEM retained susceptibility to IMP. Such intra-class carbapenem discordance is well described and reflects the agent-specific interplay between *β*-lactamase activity and outer-membrane permeability. In non-carbapenemase-producing *K. pneumoniae*, loss of the major porins OmpK35 and OmpK36 combined with overproduction of extended-spectrum *β*-lactamases or AmpC typically elevates MEM and ertapenem MICs more than IMP. In contrast, porin loss alone yields only marginal, often still-susceptible, elevations [38, 39]. Because carbapenem MICs, phenotypic carbapenemase assays and carbapenemase genotyping were not performed, we cannot determine whether the reduced MEM susceptibility observed here is carbapenemase-mediated or driven by porin loss with *β*-lactamase overproduction. The isolates are therefore best described as MEM-resistant by disc diffusion, with the underlying mechanism and true carbapenemase status undetermined.

The host-range and EOP data for Zm_01 suggest a therapeutically favourable profile. Although isolated on non-MDR Kp01, Zm_01 plated with markedly higher efficiency on MDR isolates Kp73 and Kp88, and with lower but sufficient efficiency on Kp97, echoing previous descriptions of phages that replicate more efficiently on particular MDR hosts [40, 41]. However, our EOP values are based on single experiments without technical replicates. They should be regarded as exploratory, underscoring the need for repeat assays on larger isolate panels with appropriate statistical treatment [26, 29, 30].

The plaque morphology of Zm_02 – large, clear plaques surrounded by a diffuse halo – is suggestive of phage-encoded depolymerase activity targeting capsular polysaccharides [42, 43]. Depolymerases are particularly relevant for *K. pneumoniae*, in which the capsule is a key virulence factor and contributes to antibiotic tolerance [44]. By degrading capsules and disrupting biofilm matrices, such enzymes can enhance antibiotic penetration and immune clearance [45], potentially improving outcomes in infections caused by hypervirulent, biofilm-forming MDR strains. If genomic and functional studies confirm that Zm_02 encodes a depolymerase, the phage or its enzyme could be developed as an adjunctive tool against capsule-rich *K. pneumoniae*.

A strength of this work is the demonstration that phage isolation and basic characterization can be achieved using simple adaptations of standard protocols in a low-resource setting. Logarithmic-phase cultures for enrichment were generated by manual agitation in the absence of a spectrophotometer or shaker incubator, and images of plaques and spot tests were captured with low-end smartphones rather than dedicated imaging systems. This illustrates a cost-conscious approach and shows that visually interpretable data can be obtained with widely available devices, even if image quality is inferior to that from specialized equipment. Methodologically, the final workflow represents a synthesis of diverse published protocols adapted through iterative troubleshooting in a context without local phage expertise and is intended as a template for similar laboratories.

To our knowledge, this is the first report of *K. pneumoniae* phage isolation and phenotypic characterization conducted entirely within a low-resource setting in the SADC region outside South Africa. Existing phage-related work from neighbouring countries, such as Malawi, has relied mainly on metagenomic discovery or substantial support from high-income-country laboratories [46, 47]. In contrast, the present study was performed using local infrastructure and personnel in a setting with minimal prior experience in phage biology, representing an initial step towards an in-country pipeline for *K. pneumoniae* phage discovery, optimization and eventual therapeutic evaluation.

The study has limitations. It is a single-laboratory proof-of-concept based on a small number of non-duplicate clinical isolates and a single sewer sample. Host-range testing for Zm_01 was restricted to a few *K. pneumoniae* isolates, and systematic testing for Zm_02 was not completed. Plaque counts, titres and EOP values were derived from single experiments without technical or biological replicates, reflecting constraints on time, labour and lab commodities. Identification relied on culture and biochemical tests; without MALDI-TOF or molecular typing, we could not distinguish classical from hypervirulent or specific carbapenemase-producing lineages [48]. Finally, in the absence of electron microscopy and whole-genome sequencing, we cannot yet assign formal taxonomy, define virion morphology, assess genomic safety or confirm the presence of depolymerase or other accessory functions for Zm_01 and Zm_02.

Several further constraints apply to the resistance data. Antimicrobial susceptibility was not determined for Kp97, and this isolate was not retained, precluding retrospective testing. Its resistance phenotype and MDR status are therefore unknown, and the biological relevance of Zm_01 activity against it cannot be fully interpreted. All clinical isolates derived from a single specimen type (urine) were supplied de-identified, limiting patient-level clinical context. Finally, resistance was inferred solely from disc diffusion, without MIC determination or phenotypic or molecular confirmation of carbapenemase, so carbapenem-resistance mechanisms and true carbapenemase status remain uncharacterized.

These gaps define priorities for future work: more extensive functional characterization of the phages; broader host-range mapping across a larger, well-characterized panel of MDR *K. pneumoniae*; and genomic analysis to confirm strictly lytic biology and the absence of virulence or antimicrobial-resistance genes. Such datasets will be essential for rational phage-cocktail design and for aligning any future therapeutic use with emerging regulatory expectations for clinical phages [49, 50].

This proof-of-concept was limited to *in vitro* work; as such, animal-level safety questions could not be addressed here. A recognized consideration for systemic lytic-phage therapy against Gram-negative pathogens is the release of endotoxin (lipopolysaccharide) during bacterial lysis, which can exacerbate the inflammatory and septic responses [51]. Comparative *in vitro* data indicate, however, that phage-mediated lysis may release less endotoxin than *β*-lactam killing, because phages arrest bacterial growth more rapidly [52]; endotoxin exposure can be limited further by preparing therapeutic phages in accordance with established quality and safety requirements [50]. Quantification of lysis-associated endotoxin release and the accompanying host response will therefore be an essential component of the planned *in vivo* characterization of Zm_01 and Zm_02.

More broadly, our findings support calls for increased investment in phage and phage therapy research across Zambia and the wider sub-region. Sub-Saharan Africa carries a disproportionate burden of AMR-associated mortality and is projected to experience particularly severe impacts by 2050 if current trends persist [12, 53], yet most funding for phage therapy, new antibiotics and AMR surveillance remains concentrated in high-income settings with stronger stewardship and access to novel agents [54, 55]. Because phage research can be undertaken in many existing microbiology laboratories using bacterial hosts rather than mammalian systems and high-containment facilities, building regional capacity in bacteriophage biology, genomics and therapeutics offers a realistic route to locally tailored alternatives and adjuncts to antibiotics for MDR *K. pneumoniae* and other priority pathogens. Parallel policy and regulatory initiatives will be needed to enable clinical use of phages, good manufacturing practice-grade production and clinical trials. Locally generated data, such as these preliminary findings, can help catalyse these processes and ensure that phage therapy models are adapted to low-resource, high-AMR-burden contexts rather than being imported wholesale from high-income settings.

## Supplementary material

10.1099/acmi.0.001146.v3 Supplementary Material 1.

## Abbreviations

AMC: amoxicillin–clavulanate
AST: antimicrobial susceptibility testing
CN: gentamicin
CRKp: carbapenem-resistant *Klebsiella pneumoniae*
CTX: cefotaxime
EOP: efficiency of plating
FEP: cefepime
hvKp: hypervirulent *Klebsiella pneumoniae*
IMP: imipenem
MDR: multidrug resistance
MEM: meropenem
p.f.u. ml^−1^: p.f.u. per millilitre
SAM: ampicillin–sulbactam
TE: tetracycline
TSA: tryptone soy agar
TSB: tryptone soy broth
UTH: University Teaching Hospital
VA: vancomycin
