# Supplementary material for: Lytic bacteriophages targeting carbapenem-resistant Klebsiella pneumoniae isolated in a resource-limited Zambian laboratory
Source: Access Microbiol. 2026 Sep 22;8(9):001146.v3. doi: 10.1099/acmi.0.001146.v3 (PMC13597130; doi:10.1099/acmi.0.001146.v3)
Supplement: Supplementary file 1 [file acmi-8-01146-s001.pdf]

SUPPLEMENTARY MATERIALS

1.0 TABLES

Table S1. Antimicrobial Susceptibility Profiles of Klebsiella Pneumoniae Isolates Kp01, Kp69, Kp73 and Kp88

Isolate	Antibiotic	Code	Disk content (µg)	Zone diameter (mm)	Interpretation
Kp01	Tetracycline	TE	30	21	S
Kp01	Ampicillin–sulbactam	SAM	20	17	S
Kp01	Cefotaxime	CTX	30	16	R
Kp01	Cefepime	FEP	30	18	R
Kp01	Meropenem	MEM	10	14	R
Kp01	Imipenem	IMP	30	25	S
Kp01	Amoxicillin–clavulanate	AMC	30	22	S
Kp01	Gentamicin	CN	10	20	S
Kp01	Vancomycin	VA	30	0	-
Kp69	Tetracycline	TE	30	0	R
Kp69	Ampicillin–sulbactam	SAM	20	0	R
Kp69	Cefotaxime	CTX	30	0	R
Kp69	Cefepime	FEP	30	0	R
Kp69	Meropenem	MEM	10	11	R
Kp69	Imipenem	IMP	30	24	S
Kp69	Amoxicillin–clavulanate	AMC	30	12	R
Kp69	Gentamicin	CN	10	0	R
Kp69	Vancomycin	VA	30	0	-
Kp73	Tetracycline	TE	30	21	S
Kp73	Ampicillin–sulbactam	SAM	20	13	I
Kp73	Cefotaxime	CTX	30	13	R
Kp73	Cefepime	FEP	30	8	R
Kp73	Meropenem	MEM	10	21	I
Kp73	Imipenem	IMP	30	27	S
Kp73	Amoxicillin–clavulanate	AMC	30	18	S
Kp73	Gentamicin	CN	10	9	R
Kp73	Vancomycin	VA	30	0	-
Kp88	Tetracycline	TE	30	18	S

Kp88	Ampicillin–sulbactam	SAM	20	10	R
Kp88	Cefotaxime	CTX	30	11	R
Kp88	Cefepime	FEP	30	0	R
Kp88	Meropenem	MEM	10	0	R
Kp88	Imipenem	IMP	30	25	S
Kp88	Amoxicillin–clavulanate	AMC	30	15	I
Kp88	Gentamicin	CN	10	0	R
Kp88	Vancomycin	VA	30	0	-

Abbreviations: S, susceptible; I, intermediate; R, resistant.
Source: Clinical and Laboratory Standards Institute (2023) M100 Performance standards for antimicrobial susceptibility testing. 33rd ed. Wayne, PA (CLSI supplement M100).

Table S2: CLSI M100 Disk Diffusion Breakpoints for Enterobacterales

Agent (abbrev.)	Disk content (µg)	S (≥ mm)	I / SDD (mm)	R (≤ mm)
Ampicillin–sulbactam (SAM)	20	15	12–14	11
Amoxicillin–clavulanate (AMC)	30	18	14–17	13
Cefepime (FEP)	30	25	19–24 (SDD)	18
Cefotaxime (CTX)	30	26	23–25	22
Imipenem (IMP)	10	23	20–22	19
Meropenem (MEM)	10	23	20–22	19
Gentamicin (CN)	10	18	15–17	14
Tetracycline (TE)	30	15	12–14	11
Vancomycin (VA)	30	–	–	–

Abbreviations: S, susceptible; I, intermediate; SDD, susceptible-dose dependent;R, resistant.
Source: Clinical and Laboratory Standards Institute (2023) M100 Performance standards for antimicrobial susceptibility testing. 33rd ed. Wayne, PA (CLSI supplement M100).

2.0 FIGURES

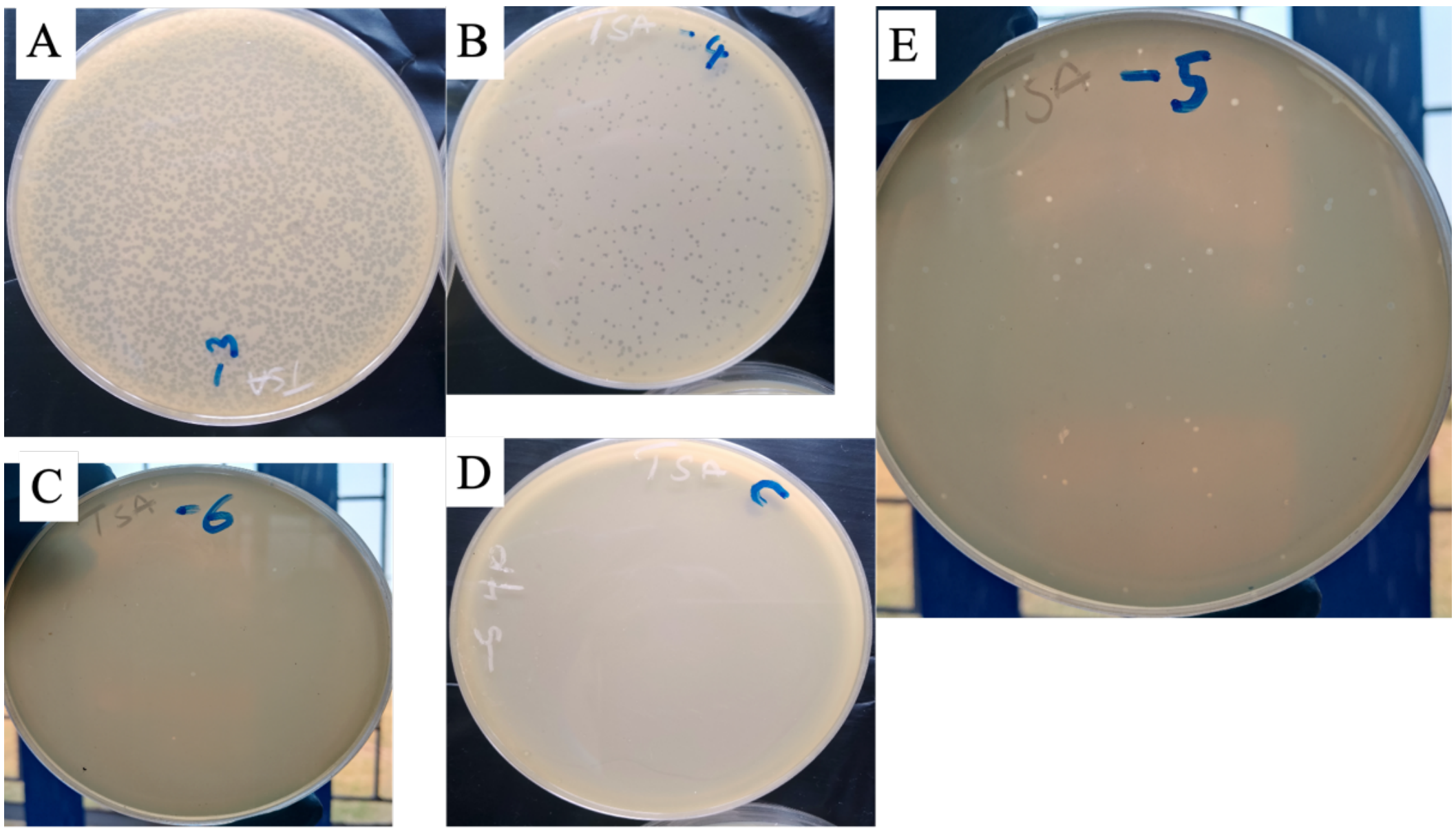

Figure S1. **Fifth-round plaque purification of *Klebsiella* phage Zm_01 on *K. pneumoniae* Kp01 using the double-agar overlay method**. Dilutions of the phage lysate were prepared in a ten-fold series and combined with the host culture before overlaying. (A) The plate with the 10^{-3} dilution shows almost confluent plaques. (B) The 10^{-4} dilution plate has a considerable number of distinct plaques. (C) The plate resulting from the 10^{-6} dilution displays only a handful of noticeable plaques. (D) The negative control plate features host bacteria without any phage added. **(E)** 10^{-5} dilution plate used to determine the phage titre; approximately 30 well-isolated plaques were counted on this plate, corresponding to an estimated concentration of 3.0×10^{7} PFU/mL for Zm_01.

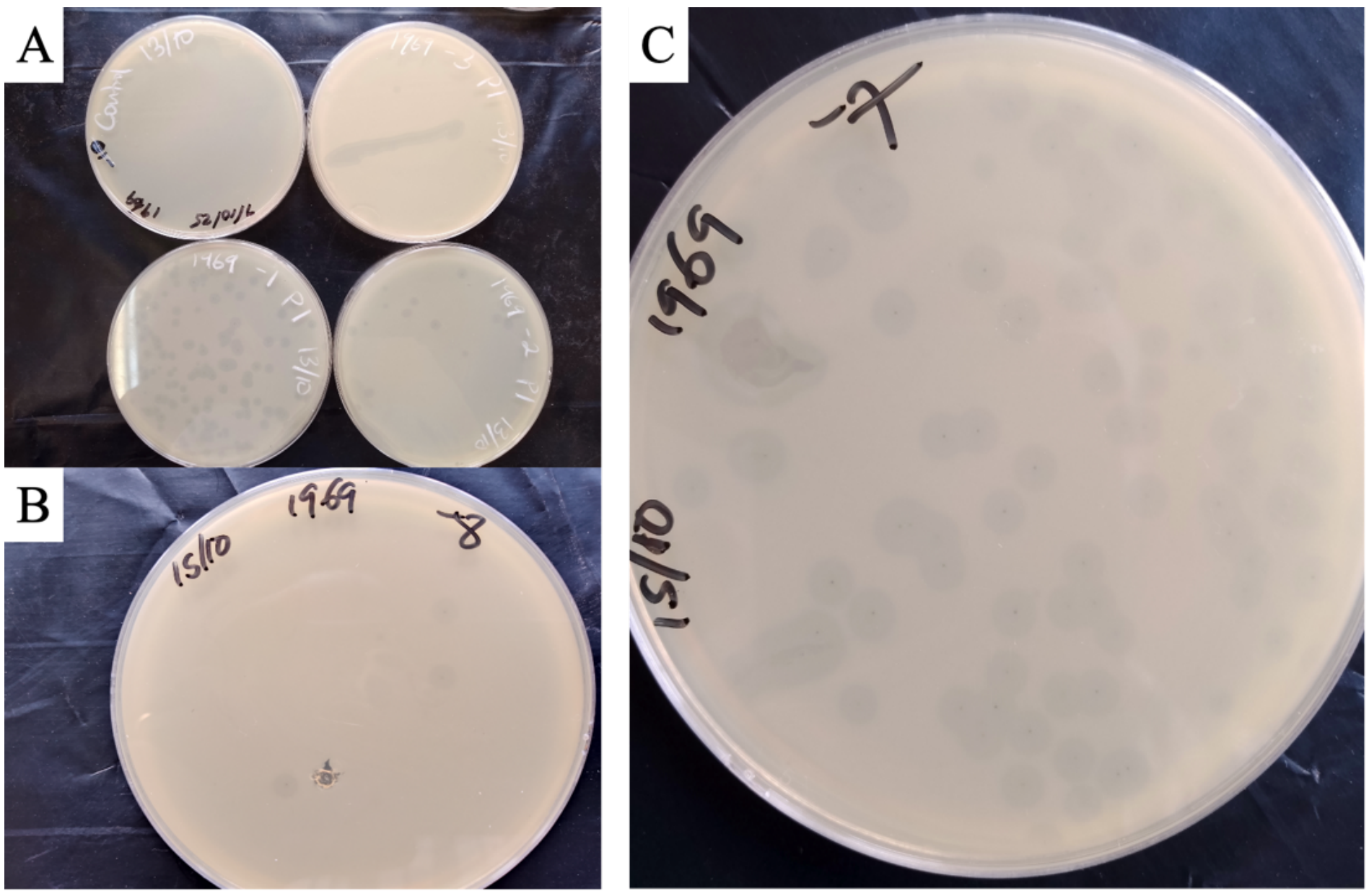

Figure S2. **Plaque assay of phage Zm_02 using multidrug-resistant *Klebsiella pneumoniae* Kp69 as host.** Phage lysates were diluted in a tenfold series and then applied using the double-agar overlay technique on TSA containing 0.4% top agar. **(A)** Control plate (no phage) alongside 10^{-1}–10^{-3} dilutions; only the phage-treated plates show discrete, Halo-like plaques on the bacterial lawn. **(B)** 10^{-8} dilution, representing the highest dilution at which plaques were still detectable. **(C)** 10^{-7} dilution plate used for titre determination; approximately 60 well-isolated plaques were counted, corresponding to a titre of 6.0×10^{9} PFU/mL.

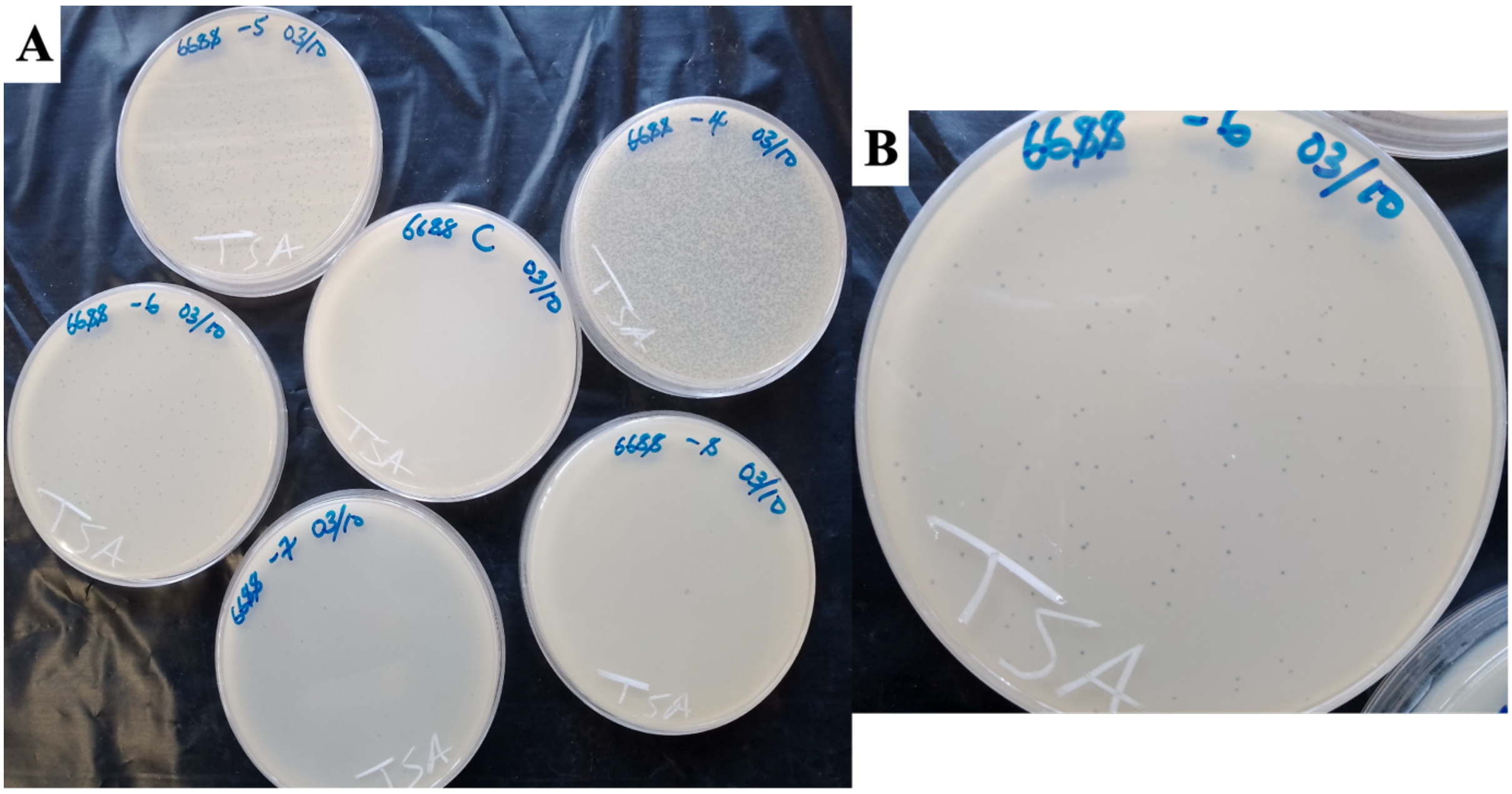

Figure S3. ***Plaque assay of phage Zm_01 on multidrug-resistant*** **Klebsiella pneumoniae** ***Kp88 for efficiency-of-plating (EOP) determination.*** *Ten-fold serial dilutions of a high-titre Zm_01 lysate were plated with logarithmic Kp88 using the double-agar overlay method on TSA.* ***(A)*** *Representative plates showing distinct plaques on Kp88 lawns across dilutions 10^{-4}–10^{-8}, alongside a phage-free control.* ***(B)*** *The 10^{-6} dilution plate, selected for enumeration, contained approximately 70 well-isolated plaques, corresponding to a Zm_01 titre of 7.0×10^{8} PFU/mL on Kp88. Using the previously determined Zm_01 titre of 3.0×10^{7} PFU/mL on the reference host* K. pneumoniae *Kp01, the calculated EOP on Kp88 is 23, i.e. ~23-fold higher plating efficiency on Kp88 than on the reference strain.*

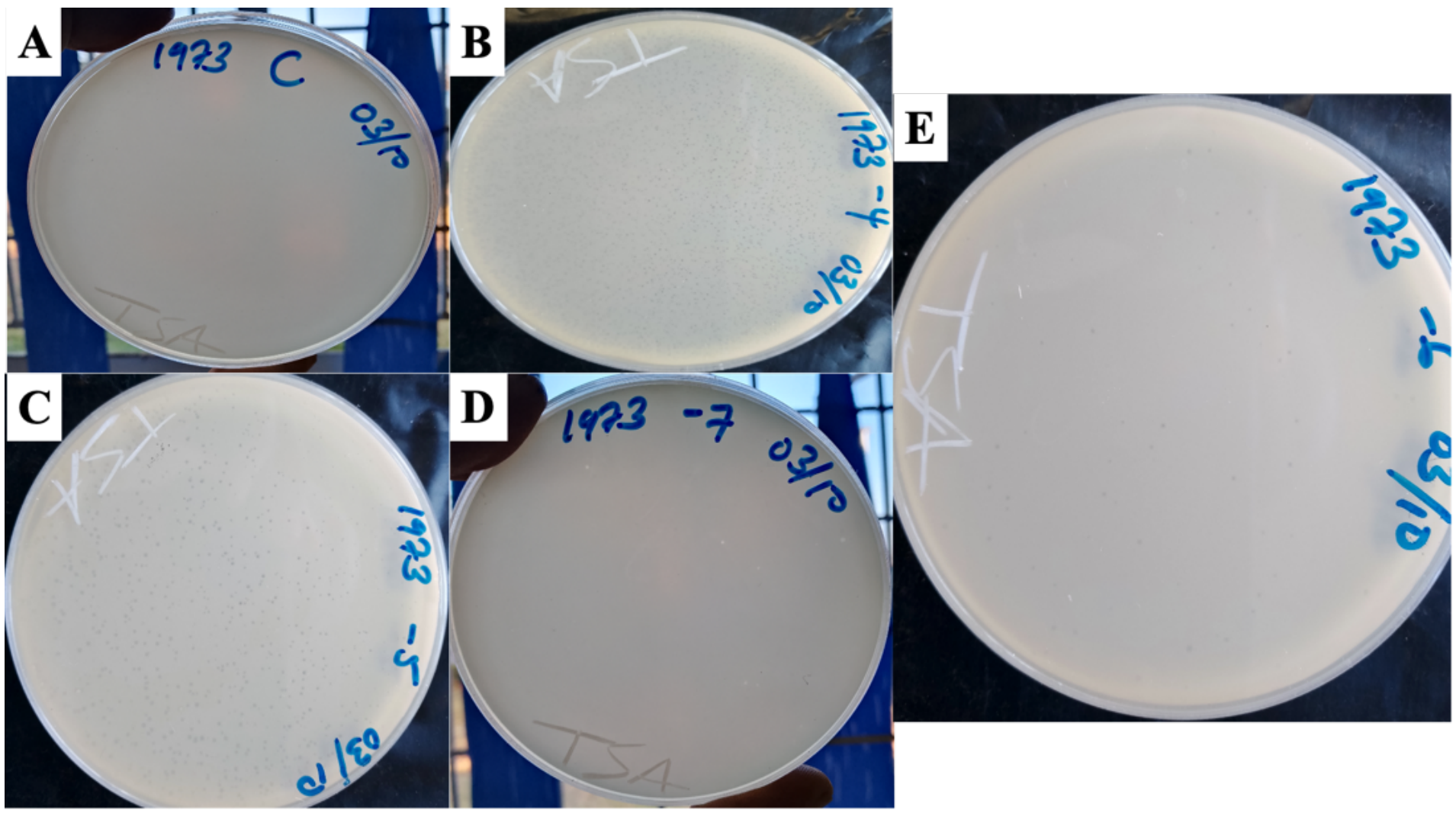

Figure S4. ***Plaque assay of phage Zm_01 on multidrug-resistant*** **Klebsiella pneumoniae** ***Kp73 for efficiency-of-plating (EOP) determination****. Ten-fold serial dilutions of Zm_01 lysate were applied to logarithmic phase Kp73 using the double-agar overlay technique on TSA plates.* ***(A)*** *The negative control without phage showed a continuous bacterial lawn with no plaques present.* ***(B)*** *The 10^{-4} dilution plate showed a nearly confluent plaque pattern.* ***(C)*** *The 10^{-5} dilution plate demonstrated a plethora of distinct plaques.* ***(D)*** *The 10^{-7} dilution represented the highest dilution at which plaques remained visible.* ***(E)*** *The 10^{-6} dilution plate was utilised for titre estimation; a total of 32 well-isolated plaques were counted, yielding a titre of 3.2×10^{8} PFU/mL for Zm_01 on Kp73. Utilising the previously established titre of 3.0×10^{7} PFU/mL for Zm_01 on the reference host K. pneumoniae Kp01, the calculated EOP for Kp73 is 11, indicating an approximate 11-fold increase in plating efficiency on Kp73 compared to the reference strain.*

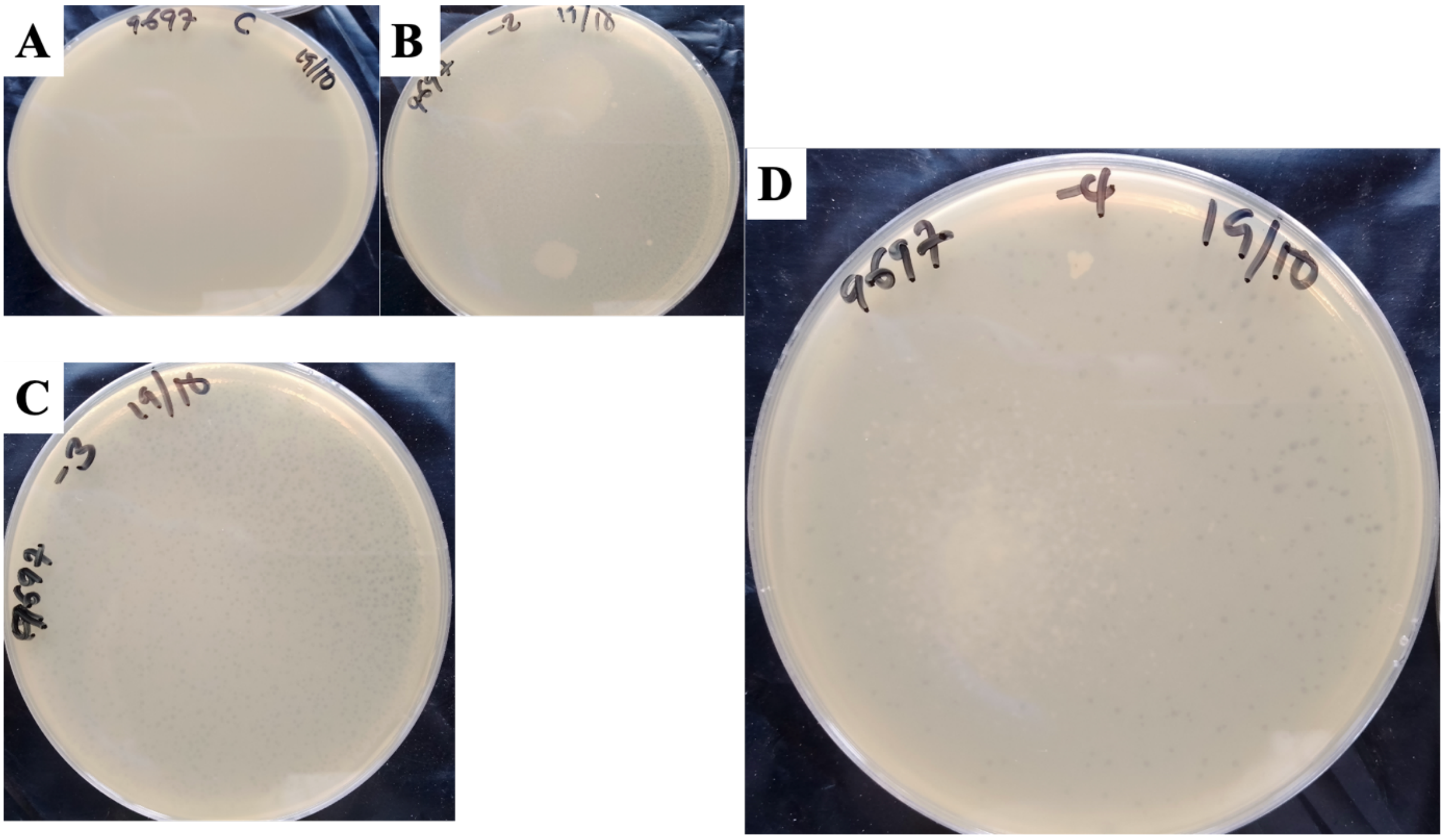

Figure S5. **Plaque assay of phage Zm_01 on multidrug-resistant *Klebsiella pneumoniae* Kp97 for efficiency-of-plating (EOP) determination.** Ten-fold serial dilutions of a Zm_01 lysate were plated with logarithmic Kp97 using the double-agar overlay method on TSA. **(A)** Phage-free negative control showing a confluent bacterial lawn without plaques. **(B)** 10^{-2} dilution plate with almost confluent lysis. **(C)** 10^{-3} dilution plate with numerous coalescing plaques. **(D)** 10^{-4} dilution, the highest dilution at which plaques were still clearly visualised; approximately 150 plaques were counted on this plate, corresponding to a Zm_01 titre of 1.5 × 10^{7} PFU/mL on Kp97. Using the previously determined Zm_01 titre of 3.0 × 10^{7} PFU/mL on the reference host *K. pneumoniae* Kp01, the calculated EOP on Kp97 is 0.5, indicating approximately two-fold lower plating efficiency on Kp97 than on the reference strain.
